# Vaccination in Whooping-Cough

**Published:** 1862-11

**Authors:** R. C. Russel


### VACCINATION IN WHOOPING-COUGH.

To the Editor of the Lancet,

SIR:—During the spring of this year, pertussis prevailed epidemically amongst children in this parish and surrounding district. As several of them had not been vaccinated, I availed myself of the opportunity of trying the effects of vaccination as a remedy for that disease.

I selected ten cases of uncomplicated whooping-cough. A few days after its employment, I found the symptoms greatly mitigated—the severity of the cough was much lessened, and the intervals between the paroxysms were lengthened. In all the cases the disease progressed favorably, and was shorter in duration than it usually is by the ordinary method of treatment.

The ages of the children varied from three months to two years, and the time of employing it was about the third week of the disease. No medicine was used in any of the cases, with the exception of gentle laxatives. Vaccination as a remedy for whooping-cough was first proposed in Germany, and is not only practised in that country, but also in America.

About thirty years ago, it was introduced into this country, and although favorably spoken of at the time, yet it was allowed to fall into disuse. Whether children so treated will enjoy an immunity from small-pox, I am unable to say; but I may mention that in three of the cases in which I attempted to revaccinate after the removal of the disease, the virus had no effect.

I am, Sir, your obedient servant,

R. C. RUSSEL, L.R.C.S.E.

—*London Lancet.*

